# Molecular Exploration of *Mycoplasma fermentans* and *Mycoplasma genitalium* in Mexican Women with Cervicitis

**DOI:** 10.3390/pathogens13111004

**Published:** 2024-11-15

**Authors:** Abraham David Bustos-López, Marcos R. Escobedo-Guerra, Marcela López-Hurtado, Jesús Roberto Villagrana-Zesati, Martha Valdés-Ramírez, Silvia Giono-Cerezo, Fernando M. Guerra-Infante

**Affiliations:** 1 Department of Microbiology, National School of Biological Sciences, National Polytechnic Institute, Prol Carpio and Plan de Ayala, Col Santo Tomás, Mexico City 11340, Mexico; davidblz612@gmail.com (A.D.B.-L.); sgiono@yahoo.com (S.G.-C.); 2 Molecular and Cellular Bioimmunology Laboratory, National Institute of Perinatology, Montes Urales 800, Col Lomas Virreyes, Mexico City 11000, Mexico; syramses@yahoo.com (M.R.E.-G.); diaclaro2000@yahoo.com.mx (M.L.-H.); 3 Gynecology and Obstetrics Department, National Institute of Perinatology, Montes Urales 800, Col Lomas Virreyes, Mexico City 11000, Mexico; dr_robertovillagrana@hotmail.com (J.R.V.-Z.); dramartha@prodigy.net.mx (M.V.-R.)

**Keywords:** *Mycoplasmataceae*, Mycoplasma, Ureaplasma, pregnancy, infertility, *Mycoplasma fermentans*, *Mycoplasma genitalium*

## Abstract

Genital Mycoplasmas are implicated in adverse pregnancy outcomes and the development of infertility. However, the role of *Mycoplasma fermentans* in these outcomes has not been adequately studied; therefore, its participation in these sufferings requires further investigation. This study aimed to evaluate the prevalence of *M. fermentans* in pregnant and non-pregnant women. End-point PCR was used to analyze two hundred and twenty-eight endocervical samples for *M. hominis*, *M. genitalium*, *M. fermentans*, *M. pirum*, *Ureaplasma urealyticum*, and *U. parvum* diagnoses. The prevalence of *Mycoplasma* spp. was as follows: *U. parvum* was found in 83 samples (36.4%), *U. urealyticum* in 39 instances (17.1%), *M. hominis* in 36 (15.7%), *M. fermentans* in 32 (14%), *M. genitalium* in 15 (6.6%), and *M. pirum* in 0 samples. No association was found between the *Mycoplasma* spp. and some infertility conditions or adverse pregnancy. However, *M. fermentans* and *M. hominis* were found to be associated with bacterial vaginosis (RR = 3.4 CI 95% 1.85–6.3, *p* < 0.005). In conclusion, *M. fermentans* and *M. hominis* were isolated more often in women with bacterial vaginosis, which suggests that these bacteria could contribute to the development of this pathology.

## 1. Introduction

*Mycoplasma genitalium* and *Mycoplasma fermentans* have been implicated in various human diseases [1,2]. Both bacteria are more difficult to culture in artificial media than other pathogenic Mycoplasmas, which contributes to their diagnosis being determined using molecular techniques [3,4]. Moreover, both *M. genitalium* and *M. fermentans* have been detected in the genitourinary tract of adults [5,6] and can, therefore, be sexually transmitted. The prevalence of *M. genitalium* infection globally in men and women in the “open population”, which refers to individuals who are not in a specific high-risk group, is about 1% to 6.4% [5]. However, the prevalence of *M. fermentans* is unknown despite it being considered a pathogenic Mycoplasma. In Mexico, the prevalence of both of the above-mentioned microorganisms has yet to be fully elucidated.

*M. genitalium* infection in women has been associated with the development of cervicitis, endometritis, salpingitis, and tubal factor infertility [5,7]. *M. fermentans* has been associated with respiratory infections, arthritis, genitourinary tract infections, and chronic fatigue syndrome and has been linked to the development of human immunodeficiency viruses [1,8,9,10]. The present study determined the prevalence of *M. fermentans, M. genitalium*, and other Mycoplasmas in women with cervicitis who attended a tertiary health institute in Mexico City. The results of this study highlight the need for further research.

## 2. Materials and Methods

### 2.1. Study Design

The study design consisted of a descriptive and cross-sectional study carried out in Mexico City.

### 2.2. Study Population

The population consisted of women between the ages of 15 and 45. All study participants were diagnosed using clinical data on endocervical infection and attended the tertiary care hospital in Mexico City for diagnosis and treatment. The study period was from September 2022 to March 2023.

### 2.3. Cervicovaginal Fluid Samples

Two hundred and forty-three cervicovaginal samples were obtained from women who attended the gynecology service at the tertiary care hospital in Mexico City. The patients met the following requirements: (a) sexual abstinence for a minimum of 72 h, (b) had not ingested antibiotics or applied vaginal suppositories at the time of sample collection, (c) presented to the sample collection well cleaned, (d) were not in their menstrual period, and (e) were not using any method of family planning. All patients signed the institutional informed consent form before participating in this study.

### 2.4. Microbiological Culture

Three swabs were obtained for each cervicovaginal sample: the first for culture on artificial media and Gram stain smear, the second for a wet mount examination, and the third for placement on a universal transport medium (UTM^®^, Copan Diagnostics, Inc., Murrieta, CA, USA). A drop of this suspension was placed between a slide and the coverslip for wet mount examination. The observation was performed using a Karl-Zeiss microscope with a 40× objective, searching for clue cells, yeast cells, pseudohyphae, and *Trichomonas vaginalis*. The smear was stained with Gram stain, and a 100× objective was used for evaluating the Nugent criteria. The artificial culture medium used was chocolate agar (Columbia CNA Agar supplemented with 5% horse or human blood). The samples were streaked using the cross-streak technique and incubated at 37 °C with 5% CO_2_ for 48 h. The presence of beta-hemolytic and grayish colonies with a diameter of 1 mm was considered to indicate possible *Gardnerella vaginalis*. Biochemical tests were performed using the Vitek Compat 2 automated system using the NH card to confirm biochemical identification.

### 2.5. Amsel Criteria and Nugent Scoring

The Amsel criteria analyzed were pH > 4.5; the presence of gray, homogeneous, and adherent vaginal discharge; the release of a fishy odor upon the addition of 10% potassium hydroxide to the secretion; and the presence of clue cells. The presence of three of the above parameters is considered to indicate bacterial vaginosis (BV). Different morphotypes were counted for the Nugent criteria, and a value was determined depending on the quantity observed. Finally, the scores were summed. If the sum was between 7 and 10, the diagnosis was BV (Supplementary Materials Table S1).

### 2.6. Mycoplasma Culture

Two hundred microliters of each sample (placed in universal transport medium) was deposited in tubes containing 0.5 mL of base medium for Mycoplasmas (BBL^®^, Becton Dickinson, Cockeysville, MD, USA) supplemented with 20% horse serum (HyClone™, Cytiva, Marlborough, MA, USA), 1% glucose (Merck^®^, Darmstadt, Germany), 3% arginine (Research Organics Inc., Cleveland, OH, USA), or 5% urea (Merck^®^). The tubes were incubated at 37 °C until the pH indicator turned alkaline.

### 2.7. Mycoplasma Strains

The reference Mycoplasma strains *U. parvum* ATCC 27815, *U. urealyticum* ATCC 27618, *M. genitalium* ATCC 33350, *M. hominis* ATCC 15488, and *M. fermentans* ATCC 19989 were used as positive controls. The reference strains were provided by the Molecular and Cellular Bioimmunology Laboratory.

### 2.8. Nucleic Acid Extraction

Nucleic acid was obtained using the phenol-chloroform technique. An amount of 200 µL of lysis regulator for white cells was added to 200 µL of the sample. Next, the sample was vortexed for 15 s and transferred to a water bath at 56 °C for 1 h. After the incubation time, 200 µL of phenol and 200 µL of chloroform were added, homogenized in a vortex for 1 min, and centrifuged at 1500× *g* for 10 min; then, the supernatant was recovered in a new Eppendorf tube. Next, 200 µL of chloroform was added to the supernatant, which was left for 15 min in the vortex and then centrifuged at 1500× *g* for 10 min. The supernatant was recovered in a new tube, and 40 µL of 1 M NaCl and 1 mL of absolute molecular biology-grade alcohol at 4 °C were added. Next, the tubes were centrifuged for 15 min at 14,500× *g*. Finally, the supernatant was decanted, and the nucleic acid was hydrated with 40 µL of DNAse- and RNAse-free water (Invitrogen by Thermo Fisher Scientific, Austin, TX, USA).

### 2.9. Mycoplasma Detection Using PCR

PCR analysis was performed on endocervical exudate samples and cultures suspected of Mycoplasmas. A reaction mixture of 12.5 µL of 2× concentrated Master mix containing Taq DNA polymerase and dNTPs (Thermo Fisher) was used. All samples were examined for the presence of *U. parvum*, *U. urealyticum*, *M. fermentans*, *M. genitalium*, *M. hominis*, and *M. pirum*, according to the described protocols of Wang [11], Grau [12], de Barbeyrac [4], and Kong [13]. Table 1 reports the characteristics of the primers of the reaction protocols used to identify each of the Mycoplasmas. The PCR products were identified on a 2% agarose gel. The gel was stained with ethidium bromide and visualized using a Multi-Image™ (Alpha Innotech Corporation, San Leandro, CA, USA) transilluminator. The Mycoplasma species were identified according to the amplification product size. Figure S1 shows the PCR products obtained from the strains used as positive controls. The PCR results were as follows: *U. urealyticum* of 443 bp, *M. hominis* of 150 bp, *M. genitalium* of 280 bp, *M. fermentans* of 209 bp, and *Ureaplasma parvum* of 403 bp.

### 2.10. Statistical Analysis

Fisher’s exact non-parametric test was used to determine the association between *Mycoplasma* infections and the categorical variables. The magnitude of the associations among the variables was expressed as the relative risk (RR) with a confidence interval (CI) of 95%. A *p*-value of less than 0.05 was considered statistically significant. SPSS statistics software for Windows, version 20.0 (IBM Corp, Armonk, NY, USA), was used for the analysis.

## 3. Results

### 3.1. Frequency of Mycoplasma spp. and Co-Infections

Two hundred and forty-three samples of cervicovaginal exudates were obtained. However, fifteen samples were eliminated because the patients had received antimicrobial treatment for a chronic Ureaplasma infection. So, two hundred and eight samples were analyzed to identify Mycoplasma strains. The results show that the prevalence of *Mycoplasma* spp. infection was as follows: *Ureaplasma parvum* was detected in 83 (36.4%) samples, followed by *U. urealyticum* in 39 (17.1%), *M. hominis* in 36 (15.7%), *M. fermentans* in 32 (14%), and *M. genitalium* in 15 (6.6%). *M. pirum* was not detected in any samples (Table 2). Other identified STI pathogens were *Chlamydia trachomatis* (2.2%), *Trichomonas vaginalis* (1.3%), and *Neisseria gonorrhoeae* (0.0%).

**Figure 1:**
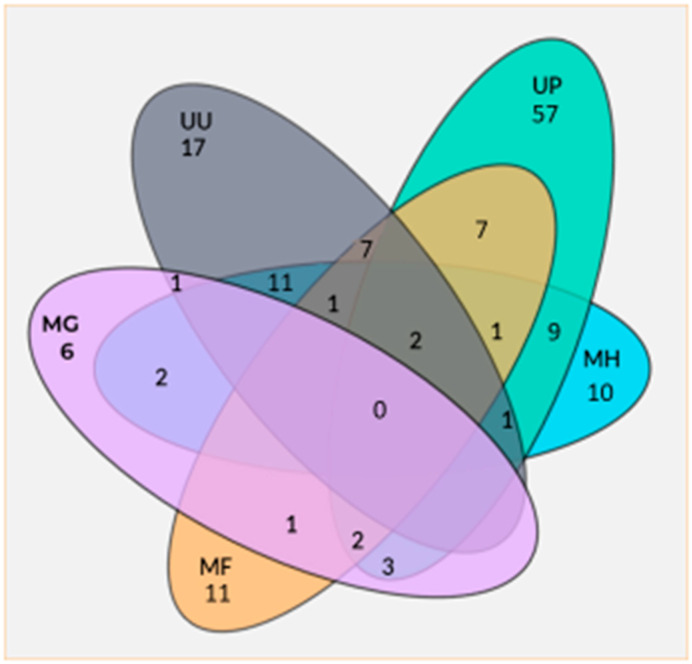
Co-infection numbers of several *Mycoplasma* spp. UU = *Ureaplasma urealyticum*; UP = *Ureaplasma parvum*; MH = *Mycoplasma hominis*; MG = *Mycoplasma genitalium*; MF = *Mycoplasma fermentans*.

A significant co-infection of *M. fermentans* and *Ureaplasma urealyticum* (RR = 2.2; CI 95% 1.14–4.3, *p* < 0.039) or *Gardnerella vaginalis* (RR = 4.7; CI 95% 2.6–8.5, *p* < 0.001) was demonstrated. Figure 1 shows several combinations of co-infections between the *Mycoplasma* spp. observed in this study.

### 3.2. Prevalence of Mycoplasma spp. Colonization in Pregnant and Infertile Women

All the participants presented some symptoms or clinical signs, such as a change in color, odor, or amount of vaginal discharge as well as itching, vaginal irritation, pain during sex or urination, and light vaginal bleeding or spotting. The age range of the patients was from 15 to 45 years, with a mean age of 30.9 ± 6.8 years. A total of 103 of the patients were women who had infertility problems, and 125 were pregnant. The prevalence percentages of *M. fermentans* and *M. genitalium* in infertile women were 10.7% and 4.9%, respectively (Table 3 and Table 4). The prevalence percentages of *U. parvum*, *M. hominis*, and *U. urealyticum* were 29.1%, 21.4%, and 18.4%, respectively (Tables S1–S3). This research highlights that the most critical cause of infertility was a uterine factor in 59.6%, followed by an endocrine–ovarian factor in 57.8%, a masculine factor in 45%, and a tubal factor infertility in 23.9%. The prevalence percentages of *M. fermentans* and *M. genitalium* in pregnant women were 16.8% and 8%, respectively. For other *Mycoplasma* spp., the prevalence results showed *U. parvum* at 42.4%, *M. hominis* at 11.2%, and *U. urealyticum* at 16%. None of the causes of infertility were associated with *Mycoplasma* spp. infections or with miscarriage, preterm birth, or newborn respiratory illness in pregnant women in this study. However, a significant colonization frequency of *M. hominis* was observed in infertile patients (RR = 1.13; CI 95% (1.0–1.27, *p* < 0.045)) (Table S4). However, in pregnant women, a significant frequency of colonization with *U. parvum* was found (RR = 1.46; 1.0–2.1, *p* < 0.04) (Table S2).

### 3.3. Bacterial Vaginosis and Candidiasis

Thirty-three cases of bacterial vaginosis were documented in this study. The results show that twenty-one of the cases were identified as a mix of *Gardnerella vaginalis* and *Mycoplasma* spp. (RR = 24.3; CI 95% 9.1–65, *p* < 0.001), by *G. vaginalis* caused eight cases, and four cases were caused by *Mycoplasma* spp. The *Mycoplasma* spp. percentages associated with *G. vaginalis* were a mixed combination as follows: 45.5% (15 cases) for *U. parvum*; 33.3% (11 cases) for *M. fermentans*; 30.3% (10 cases) for *M. hominis*; 18.2% (6 cases) for *U. urealyticum*; and 6.1% (2 cases) for *M. genitalium.* Twenty-five cases of candidiasis were documented. Of these 25 cases, *Candida albicans* caused 18, and 7 were caused by *Candida glabrata*. Interestingly, *U. parvum* (13 cases) and *M. fermentans* (six cases) were also observed in these patients.

## 4. Discussion

Mycoplasmas are considered part of the vaginal microbiome and an opportunistic pathogen. However, the conditions for one or the other case are still being elucidated. *M. fermentans* has been identified in different patients, including those who are healthy, those who are immunocompromised, and those with chronic-degenerative diseases [1,8,9]. Moreover, *M. fermentans* DNA has been detected in the lung, synovial fluid, peripheral blood, and vaginal tissues [8]. Despite the above, the pathogenesis of *M. fermentans* is not yet completely understood, and its prevalence in the genital tract is unknown. In this study, the prevalence of *M. fermentans* was 14% higher than that of *M. genitalium*, which was 6.6%, and much lower than that of *U. parvum* (36.4%). A study by Rivera A. et al. [14] demonstrated that *M. fermentans* can produce biofilm on copper intrauterine devices (T 380 A). This finding suggests that *M. fermentans* could be considered an opportunistic pathogen, mainly when using intrauterine devices.

Another pathogen is *M. genitalium*, which is generally detected in the lower urogenital tract of asymptomatic men and women, occurring in 1% to 6.4% of the general population [5,15]. However, *M. genitalium* is more prevalent in people with urogenital symptoms (sometimes significantly at more than 20%) and those attending sexually transmitted infection clinics [5,16]. In Mexico, more detailed information is required. The prevalence of *M. genitalium* in Mexico has been reported to be between 0.5% and 13.8%, depending on the type of patients studied [17,18,19]. In this study, the prevalence of *M. genitalium* was within the value reported worldwide.

Studies in infertile women have associated *M. genitalium* as the cause of pelvic inflammatory disease (PID) and tubal occlusion [5,7]. However, other studies failed to demonstrate a significant association between infection and progression to PID [5,20]. Similarly, studies have not been able to verify *M. genitalium* as a cause of tubal infertility [21,22]. In this study, only five infertile women had *M. genitalium* infection, and one of them showed tubal factor infertility. Regarding *M. fermentans*, eleven patients were infertile, and only three showed tubal occlusion. It should be noted that the sexual partners of these women showed teratozoospermia (<4%) and vacuoles in the heads of the spermatozoa (between 7 and 11%), which suggests *M. fermentans* as a possible cause of this pathology, as has been shown to occur with *M. hominis*, *U. urealyticum*, and *M. genitalium* [23,24,25].

Regarding pregnant women infected with *M. genitalium*, several studies have reported a prevalence of 1 to 4% [26,27]; however, more recent studies describe a prevalence of mostly 12 to 18% [28,29]. In this study, the prevalence result of *M. genitalium* was 7.4%. *M. genitalium* infection has been associated with preterm birth [30,31]; however, other studies have not found an association with preterm birth or pregnancy loss [32,33]. In this study, all pregnant women had deliveries at term. Moreover, in pregnant women, the prevalence of *M. fermentans* was 16.4%, and it was identified in any trimester of gestation.

*Ureaplasma* spp. is part of the vaginal microbiota and has an average colonization rate of 40 to 80% [34]. However, this bacterium has been associated with infertility, non-gonococcal urethritis, and prostatitis [35,36]. *Ureaplasma* spp. can be detected in the cervicovaginal samples of healthy and diseased women; therefore, it is considered an opportunistic pathogen. Moreover, published studies on *Ureaplasma* spp. infection in pregnant women have associated the bacterium with preterm labor, premature rupture of membranes (PROMs), and clinical chorioamnionitis [6,37,38]. The presence of *Ureaplasma* spp. in the lower genital tract has been linked to increased matrix metalloproteinases, prostaglandins, and cytokines associated with the precipitation of preterm labor and PROMs [39]. In this study, 53.5% of the participants showed colonization by *Ureaplasma* spp. The molecular identification of the species showed *U. parvum* in 36.4% and *U. urealyticum* in 17.1% of the patients.

Although the association of *U. urealyticum* with urogenital infections is well established, the role of *U. parvum* in these infections is still undetermined. The presence of *U. parvum* in the vagina of many healthy, non-pregnant women complicates understanding of the potential role of this bacterium in adverse pregnancy outcomes [38] and disorders of the reproductive system [35]. In this study, *U. parvum* was detected in 42.4% of pregnant women and 29.1% of infertile women.

*Mycoplasma hominis* can infect different parts of the female reproductive tract and lead to infertility. The prevalence of *Mycoplasma hominis* has been reported to be between 1.2% and 52% worldwide. Diverse studies are considered to be responsible for up to 10% of causes of pelvic inflammatory disease, presenting as either endometritis, pelvic adhesions, or salpingitis. In this study, the general prevalence of *Mycoplasma hominis* was 15.8%. Moreover, in infertile women, the prevalence of *Mycoplasma hominis* was 21.4%, and no association was observed with the tubal factor infertility.

In pregnant women, vaginal colonization with *M. hominis* is associated with premature rupture of membranes (PROMs), preterm delivery, and spontaneous miscarriage, and its reported prevalence is 11.2%. However, a 2021 study on South African pregnant women who were HIV-positive found a 48% prevalence rate for *M. hominis* [39]. The prevalence of *M. hominis* found in this study was 11.2%, and no association was observed in some gestation trimesters.

Bacterial vaginosis (BV) is one of the most common vaginal syndromes in infertile women. The worldwide prevalence of BV ranges from 20 to 60% [40]; in this study, the prevalence of BV was 20.4%. Other studies in Mexico have reported between 8.5 and 60% in the same Mexican population [41,42,43]. *Gardnerella vaginalis* is more frequently the etiological agent in BV; however, in this study, a mixed infection of *Gardnerella vaginalis* and *Mycoplasma* spp. was observed in 63% of BV cases, mainly with the combination of *U. parvum*, *M. fermentans*, and *M. hominis*.

Despite the above, a weakness of this study is that all the vaginal microbiota participating were not identified in patients who showed colonization by *M. fermentans* and bacterial vaginosis, which would have allowed us to make a conclusion about the participation of *M. fermentans* in this pathology.

Today, the prevalence of genital Mycoplasmas in patients with BV is higher than in those without BV. Keane FE et al. reported a 53% carriage rate of *Mycoplasma hominis* in women with BV using PCR analysis compared with women without BV, in which *Mycoplasma hominis* was undetected [44]. Lendamba et al. reported that 60.2% of women with BV were genital Mycoplasma carriers: 33.12% for *U. urealyticum*; 1.95% for *M. hominis*; and 25.11% for mixed infections (*U. urealyticum* and M. *hominis*) [45]. Although BV is treated with metronidazole (MTZ) and clindamycin, there is a treatment failure between 10% and 15% of patients one month after medication, as well as BV recurrence rates of 30% at 3 months and 50 to 80% per year after therapy with either drug [46,47]. Studies of the vaginal microbiota after MTZ treatment using metagenomics have indicated an increase in other biofilm-producing microbial populations such as *Prevotella*, *Gardnerella*, *Atopobium*, *Sneathia*, *Mycoplasma*, and *Ureaplasma* [46,48,49]. These biofilm-producing bacteria are known for their ability to adhere to surfaces and resist antibiotic treatment [50,51]; this emphasizes the need for a deeper understanding of these bacterial genera and their role in treatment failure. Therefore, detecting and identifying several *Mycoplasma* spp. and other bacterial species is essential for future studies in patients with BV. Consequently, it is advisable to provide therapeutic prescriptions based on laboratory results identifying antimicrobial resistance genes.

## 5. Conclusions

In this study, the prevalence values of *M. fermentans* and *M. genitalium* in samples from patients with vaginitis were 14% and 6.6%, respectively. Furthermore, a significant association between *G. vaginalis* and several *Mycoplasma* spp. was observed in patients with bacterial vaginosis.

## Figures and Tables

**Table 1:** Primer used and lengths of the amplified fragment.

Bacterium	Primers Set	Sequence	Gene	Molecular Size (bp)	Reference
*Ureaplasma* sp.	U4 U5	ACG ACG TCC ATA AGC AAC T CAA TCT GCT CGT GAA GTA TTA C	Urease	429	[2]
*U. parvum* *U. urealyticum*	UMS-125 UMA-226	GTA TTT GCA ATC TTT ATA TGT TTT CG CAG CTG ATG TAA GTG CAG CAT TAA ATT C	Multiband antigen	403 443	[13]
*M. fermentans*	RW004 RW005	GGA CTA TTG TCT AAA CAA TTT CCC GGT TAT TCG ATT TCT AAA TCG CCT	Element IS-like	209	[11]
*M. hominis*	MYCHOMP MYCHOMN	ATA CAT GCA TGT CGA GCG AG CAT CTT TTA GTG GCG CCT TAC	16S rDNA	150	[12]
*M. genitalium*	MgPa1 MgPa3	GAG CCT TTC TAA CCG CTG C GTG GGG TTG AAG GAT GAT TG	MgPa Adhesin	280	[4]

**Table 2:** Frequency and co-infection of *Mycoplasma* genera with other microorganisms.

Genus			*Mycoplasma fermentans*			
		*n*	Yes	No	RR CI 95%	*p*-Value
*U. parvum*	Yes	83	13	70	1.2 (0.62–2.3)	NS
	No	145	19	126		
*U. urealyticum*	Yes	39	10	29	2.2 (1.14–4.3)	0.039
	No	189	22	167		
*M. genitalium*	Yes	15	3	12	1.47 (0.51–4.3)	NS
	No	213	29	184		
*M. hominis*	Yes	36	4	32	0.76 (0.28–2.04)	NS
	No	192	28	164		
*C. trachomatis*	Yes	5	0	5	−1.7 (1.1–1.2) *	NS
	No	223	32	191		
*Candida* spp.	Yes	25	6	19	1.9 (0.86–4.1)	NS
	No	203	26	177		
*G. vaginalis*	Yes	36	15	21	4.7 (2.6–8.5)	0.001
	No	192	7	175		

NS: not significant, RR: relative risk, CI: confidence interval, and * Negative value/no association.

**Table 3:** Gynecological or obstetrical data of patients with vaginal infection by *Mycoplasma fermentans*.

			*Mycoplasma fermentans*		RR CI 95%	*p*-Value
		*n*	Yes	No		
Age	15–19	17	3	14	1.3 (0.43–3.8)	NS
	20–29	52	10	42	1.54 (0.78–3.0)	NS
	30–39	123	16	107	0.85 (0.45–1.6)	NS
	40–45	36	3	33	0.55 (0.18–1.72)	NS
Infertility		103	11	92	0.93 (0.84–1.0)	NS
Pregnant		125	21	104		
Endocrine–ovarian factor	Yes	59	9	50	3.4 (0.76–14.8)	NS
	No	44	2	42		
Tubal factor infertility	Yes	25	2	23	0.7 (0.16–2.9)	NS
	No	78	9	69		
Uterine factor	Yes	62	4	58	0.38 (0.12–1.21)	NS
	No	41	7	34		
Masculine factor	Yes	46	5	41	1.03 (0.34–3.17)	NS
	No	57	6	51		
Trimester of pregnancy	First	7	2	5	1.8 (0.51–6.1)	NS
	Second	62	10	52	0.92 (0.42–2.02)	NS
	Third	56	9	47	0.92 (0.42–2.03)	NS
Bacterial vaginosis	Yes	33	12	21	3.55 (1.9–6.55)	0.0001
	No	195	20	175		
Candidiasis	Yes	25	6	19	1.87 (0.86–4.1)	NS
	No	203	26	177		
Nugent scoring	<7	198	20	178		
	>7	30	12	18	3.96 (2.17–7.2)	0.0001

NS: not significant, RR: relative risk, and CI: confidence interval.

**Table 4:** Gynecological or obstetrical data of patients with vaginal infection by *Mycoplasma genitalium*.

			*Mycoplasma genitalium*		RR CI 95%	*p*-Value
		*n*	Yes	No		
Age	15–19	17	0	17		NS
	20–29	52	5	47	1.7 (0.61–4.7)	NS
	30–39	123	8	115	0.97 (0.37–2.6)	NS
	40–45	36	2	34	0.82 (0.2–3.5)	NS
Infertility	Yes	103	5	98	0.97 (0.9–1.04)	NS
Pregnancy	No	125	10	115		
Endocrine–ovarian factor	Yes	59	2	57	0.5 (0.087–2.9)	NS
	No	44	3	41		
Tubal factor infertility	Yes	25	1	24	0.8 (0.09–6.7)	NS
	No	78	4	74		
Uterine factor	Yes	62	2	60	0.44 (0.08–2.5)	NS
	No	41	3	38		
Masculine factor	Yes	46	0	46	−1.1 (1.04–1.14) *	NS
	No	57	5	52		
Trimester of pregnancy	First	7	0	7		NS
	Second	62	5	57	1.02 (0.31–3.3)	NS
	Third	56	5	51	1.2 (0.38–4.0)	NS
Bacterial vaginosis	Yes	33	2	31	0.91 (0.22–3.9)	NS
	No	195	13	182		
Candidiasis	Yes	25	1	24	0.58 (0.08–4.2)	NS
	No	203	14	189		
Nugent scoring	<7	198	13	185		
	>7	30	2	28	1.02 (0.24–4.3)	NS

NS: not significant, RR: relative risk, CI: confidence interval, and * Negative value/no association.

## Data Availability

The datasets used and/or analyzed during the current study are available from the corresponding author upon reasonable request.

## Supplementary Materials

The following supporting information can be downloaded at https://www.mdpi.com/article/10.3390/pathogens13111004/s1: Table S1: Morphotypes based on the Nugent criteria for bacterial vaginosis; Table S2: Gynecological or obstetrical data of patients with vaginal infection by *Ureaplasma parvum*; Table S3: Gynecological or obstetrical data of patients with vaginal infection by *Ureaplasma urealyticum*; Table S4: Gynecological or obstetrical data of patients with vaginal infection by *Mycoplasma hominis*; Figure S1: Amplicons ATCC Mycoplasma strains that were obtained through PCR using specific primers. Lane (1) 100 bp DNA ladder; Lane (2) PCR-amplicon of 429 bp of *Ureaplasma* spp; Lane (3) Amplicon of 150 bp of *M. hominis*; Lane (4) Amplicon of 280 bp of *M. genitalium*, and Lane (5) Amplicon of 209 bp of *M. fermentans*.
