# Supplementary material for: Molecular Exploration of Mycoplasma fermentans and Mycoplasma genitalium in Mexican Women with Cervicitis
Source: Pathogens. 2024 Nov 15;13(11):1004. doi: 10.3390/pathogens13111004 (PMC11597594; doi:10.3390/pathogens13111004)

Amsel criteria and Nugent scoring

The Amsel criteria analyzed in this study were vaginal pH> 4.5, presence of gray, homogeneous, and adherent vaginal discharge, release of a fishy odor upon adding 10% potassium hydroxide to the secretion, and presence of clue cells. The presence of 3 of the above parameters is considered bacterial vaginosis (BV).
Nugent criteria: different morphotypes were counted in a smear stained with the Gram technique. A value was given depending on the quantity of observed bacteria, and finally, the scores were summed. The Nugent score (Table 1) was interpreted as “negative” (score 0–3), “intermediate” (score 4–6), or “positive” (score 7–10). If the sum is between 7 and 10, it is considered dysbiosis of the vaginal microbiota or Bacterial vaginosis.

Table S1. Morphotypes based on the Nugent criteria for bacterial vaginosis

**Morphotypes seen by field**			
**Score**	**GBP**	**SGCB**	**CGVB**
0	> 30	0	0
1	1-30	< 1	1-4
2	1-4	1-4	> 5
3	< 1	5-30	-
4	0	> 30	-

GBP: Gram-positive bacillus (Lactobacillus spp); SGVB: short Gram-variable bacillus (*Gardnerella* spp, *Bacteroides*, *Prevotella*). CGVB: Curved Gram-variable Bacilli (*Mobilluncus*)

Table S2. Gynecological or obstetrical data of patients with vaginal infection by *Ureaplasma parvum*.

			Ureaplasma parvum		RR CI95%	p-Value
		n	Yes	No		
Age	15-19	17	12	5	2.1 (1.46-3.0)	0.003
	20-29	52	20	32	1.07 (0.72-1.6)	NS
	30-39	123	36	87	0.65 (0.46-0.93)	NS
	40-45	36	15	21	1.18(0.76-1.8)	NS
Infertility		103	30	73		
Pregnant		125	53	72	1.46 (1.01-2.1)	0.04
Endocrine-Ovarian Factor	Yes	59	17	42	0.98 (0.53-1.8)	NS
	No	44	13	31		
Tubal factor infertility	Yes	25	9	16	1.34 (0.71-2.53)	NS
	No	78	21	57		
Uterine factor	Yes	62	19	43	1.14 (0.61-2.14)	NS
	No	41	11	30		
Masculine factor	Yes	46	10	36	0.62 (0.32-1.19)	NS
	No	57	20	37		
Trimester of pregnancy	First	7	1	6	0.32 (0.05-2.0)	NS
	Second	62	31	31	1.43 (0.94-2.18)	NS
	Third	56	21	35	0.81 (0.53-1.23)	NS
Bacterial Vaginosis	Yes	33	16	17	1.4 (0.94-2.1)	NS
	No	195	67	128		
Candidiasis	Yes	25	13	12	1.51 (0.99-2.3)	NS
	No	203	70	133		
Nugent scoring	<7	198	68	130		
	>7	30	15	15	1.46 (0.97-2.19)	NS

NS: not significant, RR: relative risk, and CI: confidence interval.

Table S3. Gynecological or obstetrical data of patients with vaginal infection by *Ureaplasma urealyticum*.

			Ureaplasma	*urealyticum*	RR CI95%	p-Value
		n	yes	no		
Age	15-19	17	4	13	1.42 (0.57-3.5)	NS
	20-29	52	9	43	1.02 (0.52-1.99)	NS
	30-39	123	19	104	0.81 (0.46-1.44)	NS
	40-45	36	7	29	1.17 (0.56-2.444)	NS
Infertility		103	19	84	1.03 (0.91-1.16)	NS
Pregnant		125	20	105		
Endocrine-Ovarian Factor	Yes	59	12	47	1.28 (0.55-2.98)	NS
	No	44	7	37		
Tubal factor infertility	Yes	25	5	20	1.11 (0.45-2.79)	NS
	No	78	14	64		
Uterine factor	Yes	62	9	53	0.6 (0.27-1.34)	NS
	No	41	10	31		
Masculine factor	Yes	46	10	36	1.38 (0.6-3.1)	NS
	No	57	9	48		
Trimester of pregnancy	First	7	1	6	0.89 (0.14-5.7)	NS
	Second	62	11	51	1.24 (0.55-2.79)	NS
	Third	56	8	48	0.82 (0.36-1.87)	NS
Bacterial Vaginosis	Yes	33	8	25	1.53 (0.77-3.02)	NS
	No	195	31	164		
Candidiasis	Yes	25	4	21	0.93 (0.36-2.39)	NS
	No	203	35	168		
Nugent scoring	<7	198	33	165		
	>7	30	6	24	1.2 (0.55-2.62)	NS

NS: not significant, RR: relative risk, and CI: confidence interval.

Table S4. Gynecological or obstetrical data of patients with vaginal infection by *Mycoplasma hominis*.

			Mycoplasma hominis			
		n	Yes	No	RR CI95%	p-Value
Age	15-19	17	3	14	1.13 (0.39-3.3)	NS
	20-29	52	10	42	1.3 (0.67-2.5)	NS
	30-39	123	21	102	1.2 (0.65-2.2)	NS
	40-45	36	2	34	0.3 (0.08-1.25)	NS
Infertility		103	22	81	1.13 (1.0-1.27)	0.045
Pregnant		125	14	111		
Endocrine-Ovarian Factor	Yes	59	13	46	1.08 (0.51-2.29)	NS
	No	44	9	35		
Tubal factor infertility	YES	25	3	22	0.49 (0.16-1.53)	NS
	No	78	19	59		
Uterine factor	Yes	62	14	48	1.16 (0.53-2.51)	NS
	No	41	8	33		
Masculine factor	Yes	46	9	37	0.86 (0.4-1.83)	NS
	No	57	13	44		
Trimester of pregnancy	First	7	0	7		NS
	Second	62	7	55	1.02 (0.38-2.73)	NS
	Third	56	7	49	1.23 (0.46-3.3)	NS
Bacterial Vaginosis	Yes	33	12	21	2.96 (1.64-5.3)	0.001
	No	195	24	171		
Candidiasis	Yes	25	1	24	0.23 (0.03-1.62)	NS
	No	203	35	168		
Nugent scoring	<7	198	26	172		
	>7	30	10	20	2.54 (1.37-4.72)	0.012

NS: not significant, RR: relative risk, and CI: confidence interval.

Figure S1. Amplicons ATCC Mycoplasma strains that were obtained through PCR using specific primers. Lane (1) 100 bp DNA ladder; Lane (2) PCR-amplicon of 429 bp of *Ureaplasma* spp; Lane (3) Amplicon of 150 bp of *M. hominis*; Lane (4) Amplicon of 280 bp of *M. genitalium*, and Lane (5) Amplicon of 209 bp of *M. fermentans*.

Figure S1

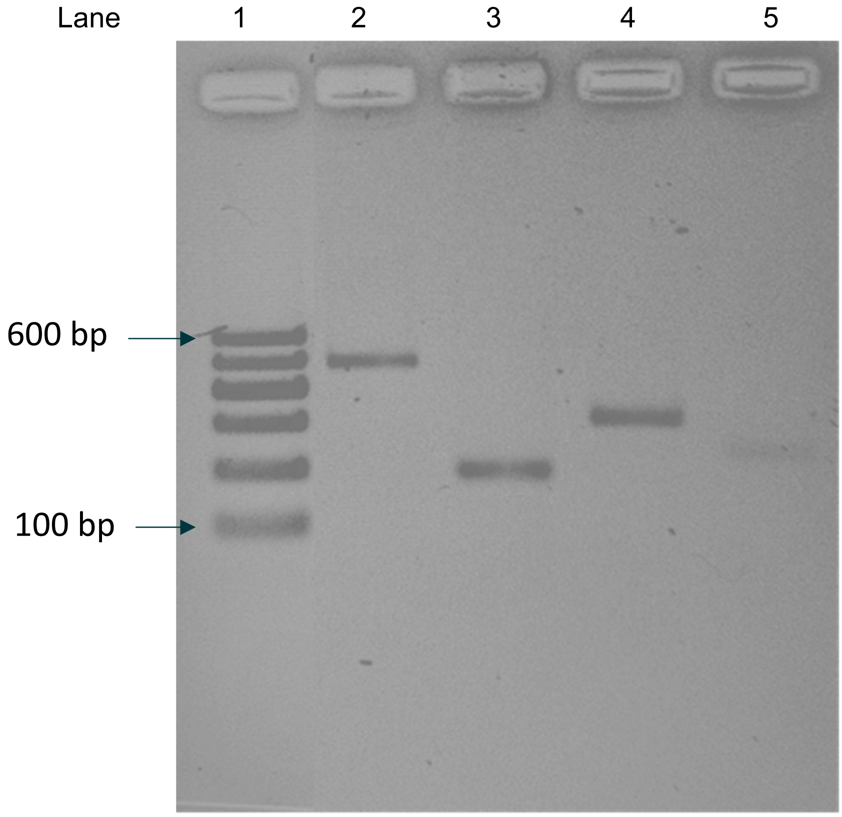

Supplement: Supplementary file 1 [file pathogens-13-01004-s001.zip › pathogens-3138756-supplementary.pdf]
